# Urine CA125 and HE4 for the Triage of Symptomatic Women with Suspected Endometrial Cancer

**DOI:** 10.3390/cancers14143306

**Published:** 2022-07-06

**Authors:** Kelechi Njoku, Chloe E. Barr, Caroline J. J. Sutton, Emma J. Crosbie

**Affiliations:** 1 Division of Cancer Sciences, School of Medical Sciences, Faculty of Biology, Medicine and Health, University of Manchester, Oxford Road, Manchester M13 9WL, UK; kelechi.njoku@manchester.ac.uk (K.N.); chloe.barr@mft.nhs.uk (C.E.B.); caroline.sutton@mft.nhs.uk (C.J.J.S.); 2 Stoller Biomarker Discovery Centre, University of Manchester, Oxford Road, Manchester M13 9WL, UK; 3 Department of Obstetrics and Gynaecology, St Mary’s Hospital, Manchester University NHS Foundation Trust, Manchester Academic Health Science Centre, Oxford Road, Manchester M13 9WL, UK

**Keywords:** endometrial cancer, biomarkers, CA125, HE4, urine, noninvasive, diagnosis

## Abstract

**Simple Summary:**

Women with suspected endometrial cancer undergo investigations that can be painful and anxiety-provoking. These tests are avoidable, as only 5–10% of symptomatic women have underlying cancer. A simple and accurate urine-based test to triage symptomatic women in the community will improve patient care. In this study, we evaluated the performance of two proteins (CA125 and HE4) for the detection of endometrial cancer in urine samples of 153 symptomatic women. We discovered that both proteins were significantly elevated in the urine of women with cancer compared to those without cancer. Urine CA125 performed better than HE4 in detecting endometrial cancer. When we combined urine CA125 and endometrial thickness measurement by transvaginal ultrasound scan, there was improvement in the detection of endometrial cancer. This is the first study to evaluate the performance of these proteins in urine for endometrial cancer detection. Confirmation in a larger sample is needed.

**Abstract:**

A simple, noninvasive and accurate detection tool that can triage women with suspected endometrial cancer for definitive testing will transform patient care. The aim of this study was to evaluate urine CA125 and HE4 levels for the detection of endometrial cancer in symptomatic women. This was a cross-sectional diagnostic accuracy study of 153 symptomatic women who underwent urgent diagnostic investigations for suspected endometrial cancer at a large gynecological cancer center. Urine samples were collected prior to routine clinical procedures. Urine CA125 and HE4 levels were determined using automated chemiluminescent enzyme immunoassays. Univariate and multivariable receiver operating characteristic (ROC) curve analyses were performed. Urine CA125 and HE4 were discovered to be significantly elevated in women with endometrial cancer, compared to controls (*p* < 0.001 and *p* = 0.01, respectively). Urine CA125 and HE4 detected endometrial cancer with an area under the ROC curve (AUC) of 0.89 (0.81, 0.98) and 0.69 (0.55, 0.83), respectively. CA125 exhibited good discriminatory potential for Type I and early-stage tumors (AUC 0.93 and 0.90, respectively). A diagnostic model that combined urine CA125 and transvaginal ultrasound-measured endometrial thickness predicted endometrial cancer with an AUC of 0.96 (0.91, 1.00). Urine CA125 displays potential as a diagnostic tool for symptomatic women with suspected endometrial cancer. When combined with transvaginal ultrasound-measured endometrial thickness, this patient-friendly, urine-based test could help triage women for invasive diagnostics or safe reassurance, reducing costs and improving patient experience.

## 1. Introduction

Endometrial cancer is the most common gynecological malignancy in high-income countries, and the sixth most frequently diagnosed cancer in women worldwide [1]. Globally, there were an estimated 417,000 incident cases and 97,000 deaths from the disease in 2020 [2]. Its incidence is rising alongside the growing obesity epidemic [3]. When diagnosed early, endometrial cancer outcomes are good, with 5-year survival estimates of 90–95% for women with International Federation of Obstetrics and Gynecology (FIGO) stage I disease [4]. However, a significant minority of women present with late-stage or biologically aggressive disease for whom 5-year survival rates are less than 20% [4,5]. Novel approaches for the early detection of endometrial cancer are urgently needed to improve outcomes for women with high-risk disease [6].

Postmenopausal bleeding is a symptom of endometrial cancer that prompts urgent investigation by a sequentially invasive diagnostic strategy, which includes transvaginal ultrasound scan, hysteroscopy, and endometrial biopsy [7,8]. This diagnostic approach exposes healthy women to unpleasant, painful, and anxiety-provoking tests that are avoidable in over 90% of cases, with huge financial implications for health service providers [6]. A noninvasive, easy-to-administer endometrial cancer detection tool that can accurately triage symptomatic women for definitive testing while reassuring the majority of women who do not have cancer would transform patient care [6,9]. At the forefront of the priorities of patients, clinicians and the general public is the development of simple, noninvasive and cost-effective tests for cancer early detection [10]. 

Cancer antigen 125 (CA125) and human epididymis protein 4 (HE4) are protein biomarkers that are associated with endometrial cancer [11,12]. CA125, a transmembrane protein and high-molecular weight mucin is known to be expressed in tubal, endocervical, and endometrial epithelium [13]. CA125 is upregulated in cancers of the breast, pancreas, and, particularly ovary, where it has found clinical utility as a diagnostic biomarker and for recurrence monitoring [14,15,16]. CA125 has also demonstrated promise as a prognostic biomarker in endometrial cancer [16]. There is growing evidence that, when used alongside HE4, CA125 may have utility for the early detection of endometrial cancer [12,17]. First identified in the epithelium of the distal epididymis, HE4, a member of the whey acidic protein family, is expressed by several tissues, including those of the female reproductive tract [18]. HE4 gene expression is upregulated in several malignancies, including those of the ovary, lung and breast [19]. While the biological function of HE4 remains unclear, its overexpression in >90% of endometrial cancers has sparked interest in its potential utility as a diagnostic biomarker for the disease [12,19]. Previous studies [12,20,21,22] have evaluated the potential diagnostic utility of one and a combination of either or both biomarkers in blood, however, their diagnostic accuracy in urine remains unreported. 

Urine is an attractive biological fluid for endometrial cancer detection as it is noninvasive, easily accessible, and can be self-collected in the community [9]. The aim of this pilot study was to evaluate the diagnostic accuracy of urine CA125 and HE4 in symptomatic women referred to secondary care for urgent investigations for suspected endometrial cancer. 

## 2. Materials and Methods

### 2.1. Research Ethics, Approvals and Patient Involvement

This study was performed in accordance with Good Clinical Practice Guidelines and the Declaration of Helsinki. Approval was by the North West Greater Manchester Research Ethics Committee (reference-16/NW/0660) with written informed consent obtained from all study participants. We developed the research question in partnership with patients, cancers, and healthcare professionals in the James Lind Alliance (JLA) Detecting Cancer Early Priority Setting Partnership (Question #1: “What simple, non-invasive, painless, cost-effective, and convenient tests can be used to detect cancer early?” [10]). This study also addresses the second-most important research priority of the JLA Womb Cancer Priority Setting Partnership (Question #2: “Which women with abnormal vaginal bleeding should be referred urgently for investigations and which can be safely reassured?” [23]).

### 2.2. Study Population

We recruited symptomatic women attending the Gynaecology Outpatient Department at St Mary’s Hospital, Manchester University NHS Foundation Trust, between April 2018 and March 2020. Cases were confirmed to have endometrial cancer based on specialist histopathological assessment of hysterectomy specimens by two gynecological pathologists reporting according to the UK Royal College of Pathology standards. Controls were women with no evidence of cancer following routine clinical diagnostics at initial presentation and followed up for any subsequent referral or presentation to our unit until October 2021. Routine diagnostics included transvaginal ultrasound assessment of endometrial thickness (ET) followed by hysteroscopy and/or endometrial biopsy for those with a thickened endometrium (>4 mm). We classed endometrial cancers according to their histological subtype (endometrioid, serous, clear-cell, carcinosarcoma) using confirmatory immunohistochemistry, and subsequently collapsed them into Bokhman’s dichotomous groupings (Types I and II). Low-grade (grade I and 2) endometrioid tumors were Type I, while high-grade (grade 3) endometrioid and nonendometrioid tumors were Type II. We used the International Federation of Gynecology and Obstetrics (FIGO) 2009 classification for disease staging. All participants provided matched urine and serum samples and clinico-pathological data, including age, BMI, diagnosis, FIGO stage, grade, and histological subtype (cases), and results of routine diagnostics including transvaginal ultrasound-measured endometrial thickness. 

### 2.3. Bio Fluid Sample Collection and Processing

Voided urine samples were self-collected prior to any clinical procedures in dry sterile collection pots. Samples were centrifuged at 1000× *g* for 10 min at room temperature and the supernatant collected and stored at −80 °C, pending analysis. Matched serum samples were collected on the same day as the urine samples and stored in 500 μL aliquots at −80 °C, pending analysis. CA125 and HE4 levels were measured using chemiluminescent enzyme immunoassays on the Fujirebio Lumipulse G600II automated analyzer (Fujirebio Europe N.V., Gent, Belgium) at the MFT Gynecological Oncology Research Facility, based on the manufacturer’s instructions. The immunoreaction cartridges used were Lumipulse^®^ G CA125-II and Lumipulse^®^ G HE4 (292631 and 234068, respectively, Fujirebio Europe N.V., Ghent, Belgium). The Lumipulse operates using a two-step sandwich immunoassay technique. The final enzyme reaction results in the generation of a luminescence signal (max wavelength 477 nm) which reflects the level of analyte in the sample and is quantified based on a calibration curve. Matched urine and serum samples were analyzed in the same batch. As CA125 and HE4 immunoassays are mainly developed for use in serum samples, we performed urine intra-assay precision analyses and evaluated the reproducibility of urine CA125 and HE4 measurements. The urine CA125 and HE4 intra-assay coefficient of variations were 15.1% and 6.2%, respectively, keeping with expected technical variability. The total inter-assay CV ranged from 5.60–7.52%. Our sample optimization steps indicated that urine samples had to be diluted to 1:100 prior to HE4 testing due to an otherwise high background. The dilution factor was ascertained based on serial 10-fold dilutions using the Lumipulse^®^ G Specimen Diluent (231180, Fujirebio Europe N.V., Ghent Belgium). Urine samples were tested undiluted for CA125. Where analyte level was at the maximum detection limit (1000 U/L) for CA125 and 1500 pmol/L for HE4), the sample was further diluted by a factor of 10 and retested. All dilutions were accounted for in subsequent concentration analysis. All samples were run in triplicate alongside manufacturer supplied quality controls to independently assess the precision of sample testing. 

### 2.4. Statistical Analyses

All statistical analyses were conducted using STATA version 16 and Graph Pad Prism version 9.3.0. Continuous data were analyzed for normality using the Shapiro–Wilk test and comparisons made by the Mann–Whitney U test. Categorical variables were compared using the Pearson’s chi-squared test or Fisher’s exact test, as indicated. Medians (±interquartile ranges) and counts (%) were reported for continuous and categorical variables, respectively. We computed the area under the receiver operating characteristic curves (AUC) and reported the exact binomial 95% confidence intervals generated using the hybrid Wilson–Brown method. The point closest to (0, 1) corner approach was used to identify optimal threshold values. Logistic regression was used to predict the probability of a cancer diagnosis based on biomarker concentrations while adjusting for confounding by age, body mass index (BMI) and type 2 diabetes mellitus (T2DM) status. The potential clinical utility of the biomarker candidates was assessed using the methodology described by Pepe and colleagues [24] by comparing the positive likelihood ratios of the biomarkers to the performance expected for clinical utility in the diagnostic setting. For this analysis, we modeled the clinical performance of the biomarkers by incorporating the prevalence of endometrial cancer and the possible cost–benefit ratio of the test using the following formula (1):

(1) $$\frac{\mathrm{TPR}}{\mathrm{FPR}}>\frac{\left(1-\;p\right)}{P}\times \;r$$

where TPR indicates true positive rate (sensitivity), FPR indicates false positive rate (1-specificity), p = prevalence of endometrial cancer in the relevant population, and r = cost–benefit ratio. The left side of the equation, also known as the positive likelihood diagnostic ratio (TPR/FPR), indicates the actual performance of the index test, while the right side of the equation indicates the performance required. If the actual test performance is greater than the required performance, the test has potential clinical utility. *p* < 0.05 was considered statistically significant for all data analyses. The study was performed in accordance with the STARD 2015 guidelines for diagnostic test reporting. 

## 3. Results

### 3.1. Clinical Characteristics of the Discovery Cohort Study Participants

**Figure 1:**
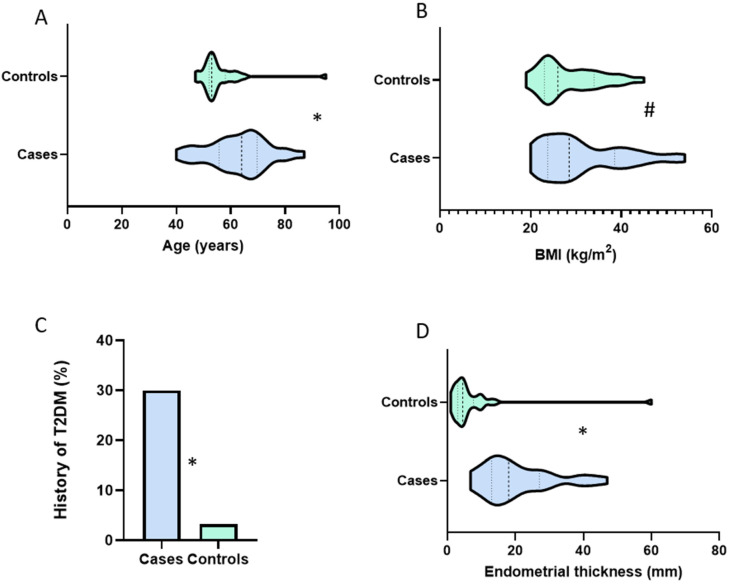
Demographic and clinical characteristics of the discovery cohort by disease status (cases: *n* = 31; controls: *n* = 30). (**A**) Age distribution; (**B**) BMI; (**C**) History of type 2 diabetes mellitus (T2DM); (**D**) Endometrial thickness. Statistical significance was determined by the nonparametric Mann–Whitney test for A, B, and D, and the chi-squared test for C. The *p*-values portrayed with * indicate *p* < 0.05 while # indicates *p* ≥ 0.05.

In total, 153 participants were included in the study. The discovery cohort comprised 61 women (30 cases and 31 controls). Their median age and BMI were 57 years (interquartile range (IQR), 52, 65) and 27 kg/m^2^ (IQR 23, 34), respectively, and they were predominantly of white British ethnicity (83.6% white, 13.1% Asian, 3.3% Black). Most of the endometrial cancers were low-grade (20, 67%), early-stage (26, 87% FIGO I/II) endometrioid (26, 87%) tumors. The symptomatic controls had abnormal vaginal bleeding which was most attributed to vulvovaginal atrophy (23.3%) or benign endometrial polyps (16.7%), but for a significant minority (43.3%), no underlying pathology was found. The demographic and clinical characteristics of the discovery cohort are portrayed in Figure 1. Women with endometrial cancer were older (median age 64 years (56, 69) vs. 53 years (52, 58), *p* = 0.001), more likely to have T2DM (9/30 vs. 1/31, *p* = 0.005) and a thicker endometrium (median ET 18 mm (13, 27) vs. 4.5 mm (3.7, 7), *p* < 0.001) compared to their control counterparts. While cases had higher BMIs than controls [median BMI 28.5 kg/m^2^ (24, 38) vs. 26 kg/m^2^ (23, 34)], this did not reach statistical significance (*p* = 0.267). There was also no statistically significant difference between cases and controls by ethnicity (*p* = 0.091), parity (*p* = 0.525), or smoking status (*p* = 0.369). Comparative analysis of CA125 and HE4 values by clinicopathological characteristics are summarized in Table S1.

### 3.2. Urine CA125 Levels in Discovery Cohort Samples

**Figure 2:**
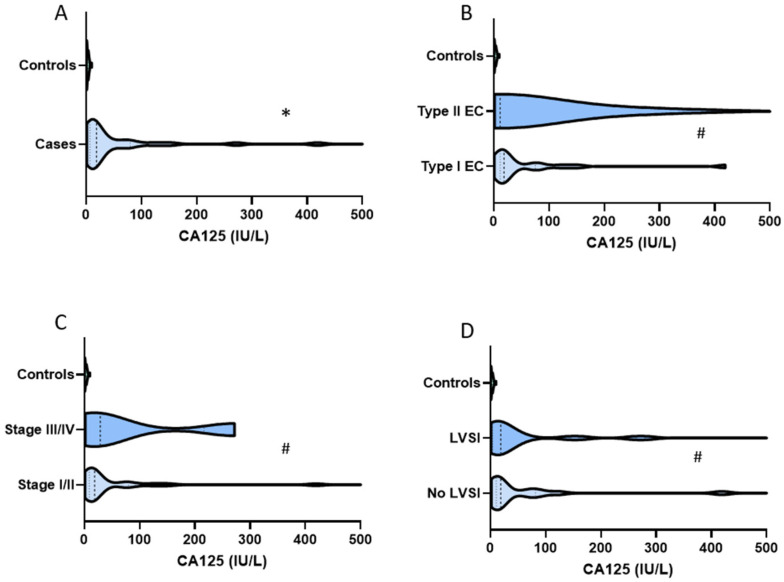
Violin plots comparing (**A**) urine CA125 by case-control status, (**B**) Bokhman group, (**C**) cancer stage, and (**D**) absence or presence of lymphovascular space invasion. Statistical significance was determined by the nonparametric Mann–Whitney U tests for relevant dichotomous groups. The *p*-values portrayed with * indicate *p* < 0.05 while # indicates *p* > 0.05.

Urine CA125 values ranged from 0.3 IU/L to 1270 IU/L with a median value of 4.8 IU/L (IQR 1.7, 18.7). There was a significant correlation between CA125 level and age (Spearman’s correlation coefficient 0.28, *p* = 0.03), and between CA125 and BMI (Spearman’s correlation coefficient 0.37, *p* = 0.003). Women aged ≥65 years had a median urine CA125 of 18.7 IU/L (IQR 13, 51.6) compared to 3.2 IU/L (IQR, 1.3, 7.5) in those <65 years (*p* < 0.001) (Table 1). Women with BMI ≥ 30 kg/m^2^ had a median urine CA125 of 16.8 (IQR 2.7, 50.2) compared to 3.5 (1.5, 9.9) in those whose BMI was <30 kg/m^2^ (*p* = 0.021) (Table 1). There was a significant correlation between urine CA125 and endometrial thickness (Spearman’s correlation coefficient 0.46, *p* < 0.001). However, at the clinical decision threshold of 4 mm, there was no evidence of a difference in urine CA125 levels in women with endometrial thickness of <4 mm (median 3.8 IU/L (IQR 0.5, 5.4) compared to those with endometrial thickness ≥4 mm (median 7.0 IU/L (IQR 1.8, 27.3, *p* = 0.06). There was also no association between urine CA125 and T2 DM status (*p* = 0.56), smoking (*p* = 0.51), or parity (*p* = 0.42). Urine CA125 levels differed significantly between endometrial cancer cases and controls, with median values of 18.7 (IQR 7.5, 77.8) and 1.9 (IQR 0.9, 4.0) respectively, *p* < 0.001 (Figure 2). There was no evidence of an association between CA125 levels and Bokhman’s endometrial cancer group (*p* = 0.61), FIGO stage (*p* = 0.85), LVSI (*p* = 0.81), or depth of myometrial invasion (*p* = 0.63) (Table 1). 

### 3.3. Urine HE4 Levels in Discovery Cohort Samples

**Figure 3:**
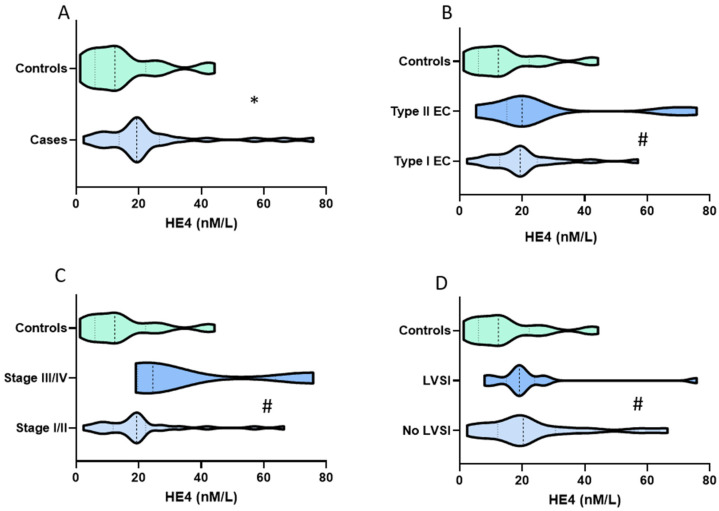
Violin plots comparing urine HE4 by case-control status (**A**), Bokhman group (**B**), FIGO stage (**C**), and absence or presence of lymphovascular space invasion (**D**). Statistical significance was determined by the nonparametric Mann–Whitney U tests for relevant dichotomous groups. The *p*-values portrayed with * indicate *p* < 0.05 while # indicates *p* ≥ 0.05.

Urine HE4 values ranged from 1.3 nm/L to 75.8 nm/L with a median value of 16 nm/L, (IQR 8.9, 22.2). There was no evidence of an association between urine HE4 and age (Spearman’s correlation coefficient 0.16, *p* = 0.23), BMI (Spearman’s correlation coefficient −0.04, *p* = 0.79), or endometrial thickness (Spearman’s correlation coefficient 0.13, *p* = 0.31). There was also no association between HE4 and T2DM status (*p* = 0.87), parity (*p* = 0.99), or smoking history (*p* = 0.70). Urine HE4 level was significantly elevated in women with endometrial cancer (median HE4 19.3 nm/L (IQR 14.1, 26.3), compared to controls (median HE4 12.4 (IQR 6, 22.2), *p* = 0.01) (Figure 3). There was no evidence of an association between HE4 levels and Bokhman group (*p* = 0.61), FIGO stage (*p* = 0.10), LVSI (*p* = 0.70), and depth of myometrial invasion (*p* = 0.50). 

### 3.4. Diagnostic Models for Endometrial Cancer Detection

**Figure 4:**
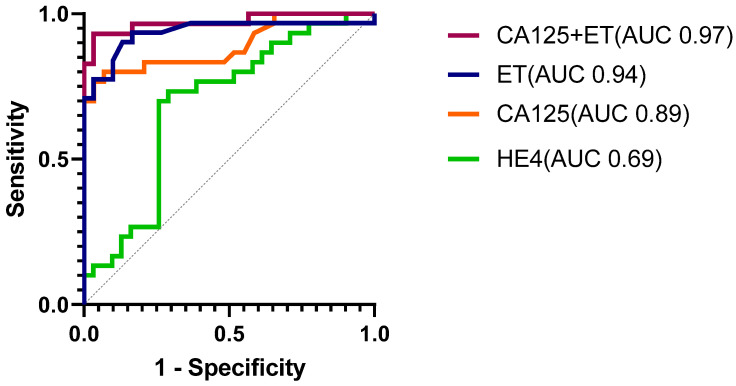
ROC curve analyses indicating the overall performance of urine CA125, HE4, endometrial thickness (ET), and a model combining CA125 and ET for the detection of endometrial cancer. AUC: Area under the curve.

We performed classical univariate and multivariate ROC curve analyses of urine CA125 and HE4 to assess their overall performance in discriminating endometrial cancers from controls. Urine CA125 and HE4 predicted endometrial cancer with AUC of 0.89 (95% CI 0.81, 0.98, *p* < 0.001) and 0.69 (95% CI 0.55, 0.83, *p* = 0.005), respectively (Figure 4). The optimal diagnostic cut-off for CA125 based on the nearest to the (0, 1) criteria was 6 IU/L, with sensitivity of 80%, specificity of 93%, and AUC of 0.87. Urine HE4, conversely, had sensitivity, specificity, and AUC values of 73%, 71% and 0.72, respectively, at its optimal diagnostic threshold of 15.2 nm/L. The addition of HE4 to CA125 did not significantly improve the diagnostic model (AUC 0.89, 95% CI 0.81, 0.98, *p* < 0.001). Transvaginal ultrasound-measured endometrial thickness demonstrated 100% sensitivity and 36% specificity at the clinically useful decision threshold of 4 mm (overall AUC 0.94, 95% CI 0.87, 1.00, *p* < 0.001). The combined model of CA125 and endometrial thickness as continuous variables was the best-performing model, improving the specificity of endometrial thickness alone, and predicted endometrial cancer with an AUC of 0.97 (95% CI 0.93, 1.00, *p* < 0.001) (Figure 4), however, the incremental benefit accruing from urine CA125 was not statistically significant (*p* = 0.447). Next, we performed multiple logistic regression analyses incorporating age, BMI, and T2DM status alongside CA125 and endometrial thickness, with the final model predicting endometrial cancer with an AUC of 0.98 (96% CI 0.95, 1.00, *p* < 0.001). The univariate and multivariate odds ratios are summarized in Table S1. 

Next, we computed positive and negative likelihood ratios for endometrial cancer detection based on urine CA125 and transvaginal ultrasound-measured endometrial thickness. At the clinically useful decision threshold of 4 mm, transvaginal ultrasound-measured endometrial thickness predicted endometrial cancer with a positive likelihood ratio of 1.56 and negative likelihood ratio of 0.0. With endometrial thickness as a continuous variable, the positive and negative likelihood ratios were 8.59 and 0.18, respectively. The combined panel of urine CA125 and endometrial thickness predicted endometrial cancer with positive and negative likelihood ratios of 12.49 and 0.15, respectively. 

**Figure 5:**
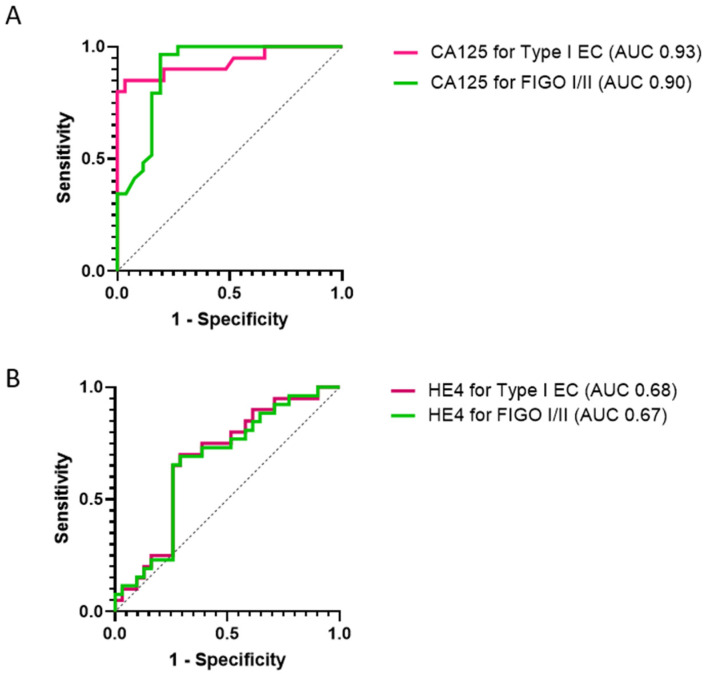
Receiver characteristic curve analyses indicating the performance of urine CA125 (**A**) and HE4 (**B**) for the detection of Type I and early-stage endometrial cancers. AUC: Area under the curve.

We conducted subgroup analyses to qualify the performance of urine CA125 and HE4 in detecting Type I and early-stage (FIGO stages I/II) endometrial tumors. Urine CA125 predicted Type I and early-stage endometrial cancers with AUC values of 0.93 (95% CI 0.85, 1.00, *p* < 0.001) and 0.90 (95% CI 0.81, 0.99, *p* < 0.001), respectively. Urine HE4 predicted Type I EC and early-stage cancers with AUC values of 0.68 (95% CI 0.53, 0.83, *p* = 0.04), and 0.67 (95% CI 0.53, 0.81, *p* = 0.03), respectively (Figure 5). Next, we assessed the performance of both biomarkers in detecting biologically aggressive and advanced-stage endometrial cancers. Urine CA125 and HE4 predicted Type II endometrial cancers with AUC values of 0.81 (95% CI 0.64, 0.99, *p* = 0.003) and 0.72 (95% CI 0.54, 0.90, *p* = 0.04), respectively, and advanced-stage (FIGO stage III/IV) tumors with AUC values of 0.84 (95% CI 0.60, 1.00, *p* = 0.03) and 0.84 (95% CI 0.69, 0.99, *p* = 0.03), respectively (Figure S1). 

### 3.5. Serum CA125 and HE4 for Endometrial Cancer Detection

Serum CA125 values for the discovery cohort ranged from 5.4 IU/L to 86.2 IU/L with a median of 17.3 IU/L (IQR 11.5, 24.5). Cases had significantly elevated serum CA125 levels (median 24.2, IQR 17.3, 36.4) compared to controls (median 13.6, IQR 10.8, 17.2, *p* < 0.001). We discovered that 8 of 30 women with cancer had elevated serum CA125 levels at the 35 IU/L reference threshold compared to 0/31 controls, *p* = 0.002. Serum HE4 levels ranged from 38.1 pmol/L to 568 pmol/L with median value of 85.3 pmol/L (IQR 70.7, 153.3). Serum HE4 values were significantly elevated in the cancers (median 152 pmol/L, IQR 93.8, 199.2) compared to controls (median 71.2 pmol/L, IQR 63.1, 84.9, *p* < 0.001). There was a statistically significant difference in the proportion of cases versus controls with elevated serum HE4 at the 77 pmol/L (25/30 vs. 12/31, *p* < 0.001) and 150 pmol/L (16/30 vs. 2/31, *p* < 0.001) reference thresholds [25].

There were significant correlations between serum and urine biomarker concentrations (correlation coefficient 0.37, *p* = 0.004 and 0.29, *p* = 0.025, respectively, for CA125 and HE4). Serum HE4 outperformed serum CA125 in predicting endometrial cancer (AUC 0.86, 95% CI 0.77, 0.96, *p* < 0.001 vs AUC 0.81 (95% CI 0.70, 0.93, *p* < 0.001) (Figure S2). The combined serum CA125 and HE4 model predicted endometrial cancer with an AUC of 0.89 (95% CI 0.80, 0.97, *p* < 0.001). Serum CA125 predicted Type I and Type II tumors with AUC values of 0.83 (95% CI 0.70, 0.96, *p* < 0.001) and 0.78 (95% CI 0.60, 0.96, *p* = 0.009), respectively. Serum HE4, however, predicted Type I and Type II tumors with AUC values of 0.82 (95% CI 0.70, 0.94, *p* < 0.001) and 0.95 (95% CI 0.87, 1.00, *p* < 0.001), respectively. Serum HE4 outperformed serum CA125 in detecting both early-stage (AUC 0.85, 95% CI 0.74, 0.95, vs 0.82, 95% CI 0.71, 0.94) and advanced-stage tumors (AUC 0.95, 95% CI 0.88, 1.00, vs. 0.73, 95% CI 0.40, 1.00).

Next, we sought to qualify the performances of CA125 and HE4 based on their expressions in both urine and serum. The combined panel of serum and urine CA125 predicted endometrial cancer with an AUC OF 0.92 (95% CI 0.84, 1.00) while the combined panel of serum and urine HE4 predicted endometrial cancer with an AUC of 0.87 (0.78, 0.97, *p* < 0.001). The model combining urine CA125 and serum HE4 predicted endometrial cancer with an AUC of 0.93 (95% CI 0.87, 1.00, *p* < 0.001). 

### 3.6. Validation of Diagnostic Performance of Urine CA125 and HE4

#### 3.6.1. Demographics of the Validation Cohort

The validation set comprised 92 symptomatic women from an independent cohort, including 33 cases and 59 controls. Their median age and BMI were 57 years (52, 73) and 28 kg/m^2^ (25, 34), respectively. The cases included women with endometrioid endometrial cancers (*n* = 20), carcinosarcoma (*n* = 3), and serous tumors (*n* = 2). Biomarker performance was also tested on a small cohort of women with severe atypical hyperplasia (*n* = 8). The controls were women with no evidence of cancer or atypical hyperplasia following routine diagnostics, and included women with benign endocervical/endometrial polyps (11.9%), uterine fibroids (10.2%), and vulvovaginal atrophy (3.4%). In the remainder of the controls, no underlying pathology was discovered. Cases were older [median age 72 years (59, 78) vs. 55 years (51, 58)], more obese [median BMI 33 kg/m^2^ (28, 37) vs. 27 kg/m^2^ (24, 33)], and had a thicker endometrium [median ET 19 mm (11, 24.4) and 3.7 mm (2.6, 6)], than their control counterparts (*p* < 0.001, 0.002, and <0.001, respectively). While the cancer cases were more likely to have T2DM compared to controls, the difference was not statistically significant (8.0% vs. 5.1%, *p* = 0.628). 

#### 3.6.2. Biomarker Performance in the Validation Cohort

**Figure 6:**
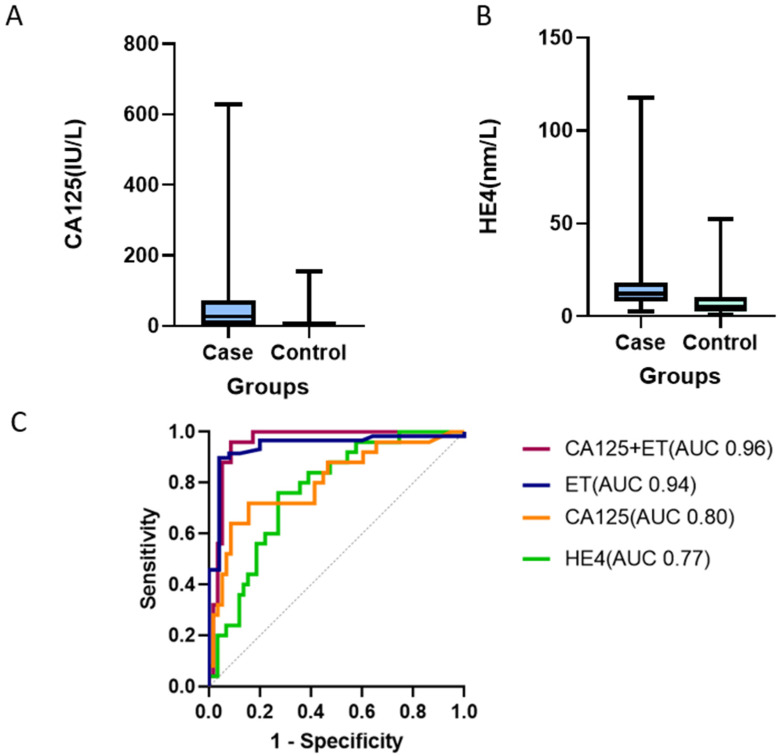
Data validation: boxplots on the distribution of CA125 (**A**) and HE4 (**B**) by cancer status. Receiver characteristic curve analyses indicating the performance of urine CA125, HE4, and the combined CA125 and endometrial thickness model, in the validation cohort (**C**). AUC: Area under the curve.

Urine CA125 concentration was confirmed to be significantly elevated in women with endometrial cancer compared to controls (median CA125 27.2 IU/L, IQR 4.6, 70.6) vs. 2.5 IU/L (IQR 1.0, 7.9, *p* < 0.001). Similarly, urine HE4 was higher in the cancer cases than the controls (median HE4 12 nm/L (IQR 9.1, 16.8) vs. 5.2 nm/L (IQR 2.6, 10.5, *p* < 0.001) (Figure 6). Urine CA125 and HE4 discriminated cancers from controls with AUC of 0.80 (95% CI 0.70, 0.91), *p* < 0.001) and 0.77 (95% CI 0.67, 0.87, *p* < 0.001), respectively (Figure 6). When we applied the individual decision thresholds derived from the main study cohort, urine CA125 predicted endometrial cancer with a sensitivity of 72% (95% CI 52,86) and specificity of 71% (95% CI 58,81), while urine HE4 predicted endometrial cancer with a sensitivity of 32% (95% CI 17.2, 51.6) and specificity of 88% (95% CI 77.5, 94.1). The model combining urine CA125 and endometrial thickness (the best-performing model in the discovery cohort) also indicated excellent discriminatory potential in the validation cohort, with an AUC of 0.96 (95% CI 0.91, 1.00, *p* < 0.001). Neither urine CA125 nor HE4 significantly discriminated women with atypical hyperplasia from controls [AUC 0.60 (0.40, 0.79), *p* = 0.371 and 0.50 (0.31, 0.70), *p* = 0.985], respectively. 

#### 3.6.3. Potential Clinical Utility of Urine CA125 in the Diagnostic Setting

Finally, we sought to describe the potential clinical utility of urine CA125 for the detection of endometrial cancer in a real-world setting. Based on the methodology described by Pepe and colleagues [24], we modeled the clinical utility of urine CA125 in a diagnostic setting. For this analysis, the *p* was set as 5% (expected prevalence of endometrial cancer in symptomatic women) while r was set at 0.1, the estimated value for 10 patients being subjected to unnecessary tests for every cancer diagnosed. The positive likelihood diagnostic ratio for urine CA125 was 11.4, exceeding the required performance value of 1.9, and confirming the potential clinical utility of urine CA125 for endometrial cancer detection in symptomatic women. 

## 4. Discussion

### 4.1. Main Findings

In this study, we demonstrate evidence for the potential utility of urine CA125 as a triage tool for endometrial cancer. Urine CA 125 indicated high specificity for the exclusion of endometrial cancer, and when used alongside transvaginal ultrasound-measured endometrial thickness, predicted endometrial cancer with an AUC of 0.96. Our data suggest that urine CA125 could reduce the number of symptomatic postmenopausal women undergoing invasive intrauterine investigations that are unwarranted. Moreover, urine CA125 may have clinical utility in a real-world setting based on the methodology described by Pepe and colleagues [24]. Confirmation of these findings in an independent cohort is warranted. 

### 4.2. Strengths and Limitations

To our knowledge, this is the first study exploring the potential utility of urine CA125 and HE4 for the detection of endometrial cancer in symptomatic women. The exploitation of urine for cancer detection, the prototype noninvasive sample, is a major strength of our study, as it is a highly acceptable sample type to women and offers the potential for community-based collection [9]. The performance of the biomarker candidates was tested in an independent validation cohort, thus minimizing the inflation in performance that characterizes biomarker studies that fail to do so. Our control group comprised symptomatic women with no evidence of cancer and constitutes the ideal comparator group for endometrial cancer biomarker studies as these are the women for whom the new test is intended [26]. All controls were excluded as cancer cases using standard endometrial cancer diagnostic pathways and followed up with for a period of 12 months, thus alleviating concerns about misclassification bias. Urine CA125 can be easily isolated and quantified using high-throughput methods that are available in every laboratory, with no expertise required and at a low cost, thus enhancing its translational potential. 

Limitations of our study include the relatively small sample size and lack of data on the molecular subtypes of endometrial cancer, which would have allowed for an exploration of the performance of urine CA125 and HE4 for the detection of specific endometrial cancer molecular phenotypes. CA125 is a nonspecific marker that may be elevated in other conditions including liver disease, pelvic inflammatory disease, benign gynecological conditions such as endometriosis, and uterine fibroids, limiting its specificity [27]. We do not know how well urine CA125 will perform in asymptomatic women or premenopausal women with a genetic predisposition to endometrial cancer, for example Lynch syndrome. Our study participants were mostly of white British ethnicity and further studies are needed to confirm the diagnostic utility of urine CA125 in other populations. Urine as a source of protein biomarkers may be influenced by several confounding factors such as hydration status, renal function, medications used, and more. Further work is needed to standardize urine sample collection and processing and to check the performance of urine CA125 in a much larger population of symptomatic women before it can be introduced as an endometrial cancer triage tool in routine clinical practice. Furthermore, there is wide variability in the analytical immunoassay platforms used in various research studies, especially for HE4 [28]. There are several HE4 isoforms and available anti-HE4 antibodies that recognize different HE4 epitopes. HE4 assays measure total HE4 levels and are unable to distinguish the various proteins encoded by different mRNA variants [29]. As such, further work is needed to characterize and harmonize putative biomarker assays before clinically useful diagnostic protocols can be established.

### 4.3. Interpretation

Few studies have explored the diagnostic utility of urine CA125 and HE4 for the detection of gynecological malignancies [30,31,32,33,34]. None have yet been conducted in the context of endometrial cancer. Moore and colleagues investigated the utility of urine CA125 for ovarian cancer detection and reported an AUC of 0.73, 33% sensitivity, and 90% specificity in women presenting with adnexal masses [30]. The addition of serum CA125 and HE4 in the diagnostic model improved the biomarker performance with AUC values of 0.80 and 0.90, respectively [30]. Hellstrom and colleagues were the first to assess the utility of urine HE4 for the detection of ovarian cancer [31]. In a retrospective study of 36 healthy women and 79 women with ovarian cancers, they demonstrated that urine HE4 detects ovarian cancer with 94.4% specificity, including 86.6% of stage I/II cancers and 90.5% of serous ovarian tumors [31]. Further data from Wang and colleagues explored the diagnostic utility of urine HE4 using a microchip ELISA-based cell phone-coupled device and reported both a sensitivity and specificity of 90%, respectively, for ovarian cancer detection [32]. These findings align with other previously published data [33,34,35,36,37] where urine HE4 has demonstrated potential as a diagnostic biomarker for ovarian cancer. A meta-analysis of 413 ovarian cancer patients and 573 controls concluded that urine HE4 has potential as a diagnostic biomarker in ovarian cancer, but was limited by marked heterogeneity of included studies with respect to diagnostic thresholds, in addition to small sample sizes [38].

In our study, urine CA125 outperformed urine HE4 for endometrial cancer detection, and when used alongside transvaginal ultrasound-measured endometrial thickness, demonstrated good accuracy for clinical translation. The incremental benefit accruing from urine CA125 was not statistically significant and was almost certainly limited by small numbers. The specificity of CA125 at its optimal diagnostic threshold (93%) compares favorably to the transvaginal ultrasound scan, whose specificity at the clinically useful endometrial thickness cutoff value of 4 mm is just 46% [39].Testing urine CA125 in symptomatic postmenopausal women whose endometrial thickness exceeds the established clinical threshold of 4 mm could therefore reduce the number of women undergoing invasive intrauterine investigations, specifically hysteroscopy and/or endometrial biopsy that are unwarranted, although the effect size is small. Urine CA125 thus has potential to complement a transvaginal ultrasound scan as a triage tool in the diagnostic work-up of symptomatic women, and studies exploring its potential clinical utility are needed. If validated in a larger independent cohort, women with an endometrial thickness >4 mm plus a positive urine CA125 test could be referred for definitive testing, while those with negative results are reassured without the need for unpleasant, painful, and anxiety-provoking tests, with large cost savings for health service providers. An endometrial cancer risk algorithm that combines urine CA125 and endometrial thickness as continuous variables may serve as a useful risk-assessment tool in symptomatic postmenopausal women in the community. Urine CA125 may also have a role in screening high-risk women, for example, those taking tamoxifen or those with Lynch syndrome, and prospective studies exploring this possibility are urgently needed.

Urine CA125 indicated potential for the detection of early-stage tumors and outperformed both serum CA125 and HE4. The early detection of endometrial cancer improves outcomes and enables conservative management options to be offered to women who wish to retain their uterus for fertility-sparing reasons [6]. Previous studies have demonstrated that elevated serum CA125 and HE4 may be indicative of aggressive endometrial cancer phenotypes [40]. Serum CA125 has been reported to predict the presence of lymphovascular space invasion, deep myometrial invasion, and metastatic disease [41,42,43,44]. HE4, however, has demonstrated potential in identifying high-risk endometrial cancer and may predict response to treatment with levonorgestrel-releasing intrauterine system in low-grade early stage endometrial cancer [45,46]. We were unable to explore the prognostic utility of these markers due to low overall numbers and short duration of follow up.

## 5. Conclusions

In conclusion, when used in symptomatic postmenopausal women with a thickened endometrium, urine CA125 has potential to identify a subset of women without sinister underlying pathology, for whom hysteroscopy and endometrial biopsy can be safely withheld. Given its simplicity, low cost, ease of collection, and acceptability to patients, urine CA125 could assist the investigation of women presenting with symptoms in whom endometrial cancer must be ruled out.

## Figures and Tables

**Table 1:** Urine CA125 and HE4 levels in women with endometrial cancer and symptomatic controls (discovery cohort).

		CA125 (IU/L)		HE4 (nm/L)	
Clinico-Pathological Characteristics	N (%)	Median (IQR)	*p*-Value	Median (IQR)	*p*-Value
**Age**					
<65 years	44 (72.1%)	3.2 (1.3–7.5)	<0.001	14.2 (7.8, 22.3)	0.1203
≥65 years	17 (27.9%)	18.7 (13–51.6)		19.3 (14.1, 21.6)	
**BMI (kg/m^2^)**					
BMI < 30	35 (57.4%)	3.5 (1.5–9.9)	0.021	17.5 (8.0, 22.4)	0.731
BMI ≥30.0	26 (42.6%)	16.8 (2.7–50.2)		14.3 (9.2, 22.2)	
**Disease status**					
Cancer	30 (49.2%)	18.7 (7.5, 77.8)	<0.001	19.3 (14.1, 26.3)	0.011
Control	31 (50.8%)	1.9 (0.9, 4.0)		12.4 (2.6, 22.2)	
**Histology**					
Type I EC	20 (66.7%)	18.7 (12.3, 72.4)	0.611	19.3 (13.2, 23.3)	0.610
Type II EC	10 (33.3%)	11.9 (1.7, 271.9)		20.0 (17.5, 27.5)	
**Stage**					
FIGO I/II	26 (86.7%)	18.7 (9.9, 77.8)	0.848	19.3 (12.4, 20.8)	0.105
FIGO III/IV	4 (13.3%)	29.0 (4.0, 161.7)		24.6 (20.3, 51.7)	
**LVSI**					
No	17 (56.7%)	18.7 (11.6, 77.8)	0.812	20.3 (14.1, 28.0)	0.703
Yes	13 (43.3%)	18.7 (6.3, 51.6)		19.1 (17.5, 21.6)	
**Myometrial invasion**					
<50%	17 (56.7%)	18.7 (11.6, 77.8)	0.628	19.3 (12.4, 20.8)	0.502
>50%	13 (43.3%)	18.7 (6.3, 51.6)		19.3 (18.8, 27.5)	

## Data Availability

Data are available through the corresponding author upon reasonable request.

## Supplementary Materials

The following supporting information can be downloaded at: https://www.mdpi.com/article/10.3390/cancers14143306/s1, Figure S1: ROC curse analysis of urine CA125 and HE4 for (A) the detection of Type II endometrial cancer and (B) advanced stage (FIGO III/IV) disease; Figure S2: ROC curve analyses of serum CA125 and HE4 for the detection of endometrial cancer. Table S1: Diagnostic performance of urine CA125 and HE4 levels based on logistic regression in the discovery cohort.
