# Supplementary material for: Urine CA125 and HE4 for the Triage of Symptomatic Women with Suspected Endometrial Cancer
Source: Cancers (Basel). 2022 Jul 6;14(14):3306. doi: 10.3390/cancers14143306 (PMC9313438; doi:10.3390/cancers14143306)
Supplement: Supplementary file 1 [file cancers-14-03306-s001.zip › cancers-1766993-supplementary.pdf]

cancers

MDPI

Article

Urine CA125 and HE4 for the Triage of Symptomatic Women with Suspected Endometrial Cancer

Kelechi Njoku, Chloe E. Barr, Caroline J. J Sutton and Emma J. Crosbie

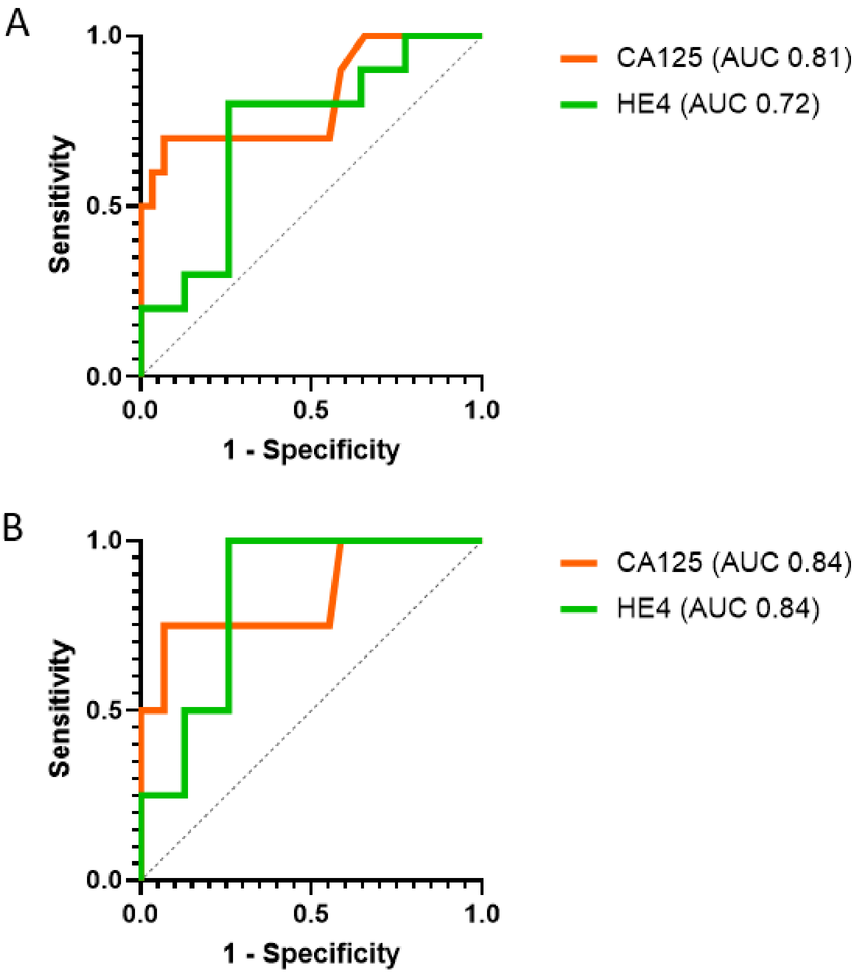

Figure S1. ROC curse analysis of urine CA125 and HE4 for (**A**) the detection of Type II endometrial cancer and (**B**) advanced stage (FIGO III/IV) disease.

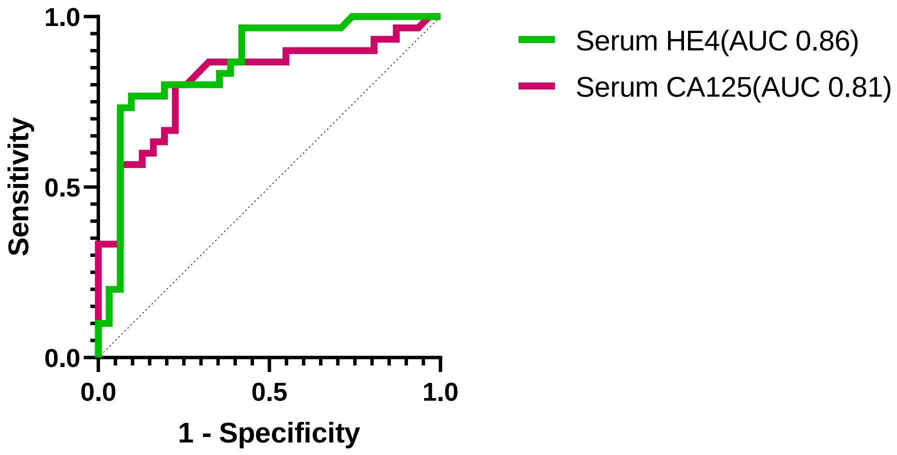

Figure S2. ROC curve analyses of serum CA125 and HE4 for the detection of endometrial cancer.

Table S1. Diagnostic performance of urine CA125 and HE4 levels based on logistic regression in the discovery cohort.

Model	Prediction	Crude OR (95%CI)	Crude *p*-value	Adjusted OR (95%CI)	Adjusted *p*-value
**Based on optimal diagnostic thresholds**					
**CA125**	Disease status	27(6.8,107.3)	<0.001	42(7.2,247)	<0.001
**HE4**		6.7(2.2,20.6)	0.001	8.9(2.3,35.1)	0.002
**Based on CA125 and HE4 as continuous data**					
**CA125**	Disease status	1.4(1.1,1.7)	0.002	1.5(1.2,1.9)	0.002
**HE4**		1.05(1.0,1.09)	0.04	1.05(1.00,1.07)	0.049

Adjusted models include age, BMI and T2DM status.
